# The influence of storage and heat treatment on a magnesium-based implant material: an in vitro and in vivo study

**DOI:** 10.1186/s12938-015-0091-8

**Published:** 2015-10-19

**Authors:** Katja Bracht, Nina Angrisani, Jan-Marten Seitz, Rainer Eifler, Andreas Weizbauer, Janin Reifenrath

**Affiliations:** Small Animal Clinic, School of Veterinary Medicine Hannover, Bünteweg 9, 30559 Hannover, Germany; Department of Materials Science and Engineering, Michigan Technological University, 1400 Townsend Dr, Houghton, MI 49931 USA; Institute of Material Science, Leibniz University Hannover, An der Universität 2, 30823 Garbsen, Germany; Hannover Medical School, CrossBIT, Center of Biocompatibility and Implant-Immunology, Feodor-Lynen-Straße 31, 30625 Hannover, Germany

**Keywords:** Magnesium, Degradable, Storage, In vitro, In vivo

## Abstract

**Background:**

Magnesium alloys are recommended as a potential material for osteosynthesis. It is known that storage-induced property modifications can occur in materials like aluminum. Thus the aim of this study was to analyze the influence of storage durations of up to 48 weeks on the biomechanical, structural, and degradation properties of the degradable magnesium alloy LAE442.

**Methods:**

Extruded implants (n = 104; Ø 2.5 mm × 25 mm) were investigated after storage periods of 0, 12, 24, and 48 weeks in three different sub-studies: (I) immediately after the respective storage duration and after an additional (II) 56 days of in vitro corrosion in simulated body fluid (SFB), and (III) 48 weeks in vivo corrosion in a rabbit model, respectively. In addition, the influence of a T5-heat treatment (206 °C for 15 h in an argon atmosphere) was tested (n = 26; 0 week of storage). Evaluation was performed by three-point bending, scanning electron microscopy, radiography, µ-computed tomography, evaluation of the mean grain size, and contrast analysis of precipitations (such as aluminum or lithium).

**Results:**

The heat treatment induced a significant reduction in initial stability, and enhanced the corrosion resistance. In vivo experiments showed a good biocompatibility for all implants. During the storage of up to 48 weeks, no significant changes occurred in the implant properties.

**Conclusions:**

LAE442 implants can be safely used after up to 48 weeks of storage.

## Background

Degradable magnesium implants have been recommended as a potential material for osteosynthesis, as they have shown positive results in a number of research studies [1]. An essential step prior to using implants in patients is the performance of tests according to various specific ISO standards (for example, ISO 13485 [2]; ISO 14155 [3]). For quality assurance of a degradable implant it is necessary to ensure that the properties of the material remain constant over a defined period of time. The outage of titanium implants for example is determined by the end of the sterility [4]. Edlund et al. [5] analyzed the influence of storage on polymeric degradable implants for up to 5 months and one of the effects they observed was an increase in hydrolysis on the implant surface. However, studies with analyses focusing on the influence of different storage durations on magnesium-based implants are rare. Changes in the characteristics of two different magnesium containing alloys (AZ91D and AM60) and of pure magnesium were tested in a “humidity chamber” with different conditions of temperature, relative humidity, and storage durations (4–11 days) [6]. The results showed that high relative humidity and low temperature (<100 °C) encouraged corrosion of the implant surface. Ullman et al. [7] showed that with increasing storage duration of LANd442 pins, there was an increase in the number of oxygen enriched regions on the implant surface, in the mean grain size of the metal, and in the levels of precipitations (such as aluminum/lithium or rare earth elements).

Various studies have dealt with the influence of heat treatment on the properties of degradable magnesium-based implants [8–11]. A carefully chosen heat treatment can enhance corrosion resistance [12, 13]. The alloy LAE442 showed preferable results in previous studies, but we could not find any studies in the literature focusing on the influence of different storage durations up to 48 weeks. Thus, the objective of this work was to investigate the influence of storage durations of up to 48 weeks using in vitro and in vivo techniques.

## Methods

### Implant material

For this study, cylindrical LAE442 implants (2.5 mm × 25 mm, n = 130) were produced by die-casting and subsequent direct extrusion, as described by Seitz et al. [14]. The exact composition of this alloy was analyzed by ICP-OES immediately after fabrication (besides magnesium 3.7 mg/l lithium, 3.62 mg/l aluminum and 1.27 mg/l rare earths). The samples were packed into sterile bags individually, underwent a gamma sterilization (25 kGy of cobalt-60 radiation; Rüsch Sterilisationsservice GmbH, Kernen, Germany), and were randomly divided into five groups (n = 26). The respective durations of storage (all in dark, dry, room temperature conditions) were 0, 12, 24, and 48 weeks as well as 0 week with an additional heat treatment. The respective samples were heat treated in an argon atmosphere at 206 °C for 15 h with subsequent cooling of the samples at room temperature according to standard T5 heat treatment protocols. A short-term and low temperature T5-procedure was chosen in order to optimize the alloy’s tensile properties while keeping its as extruded texture.

Within this study the materials properties and conditions were analyzed in three different states (sub-studies): (I) immediately after the respective storage duration, (II) after subsequent in vitro corrosion of 56 days, and (III) after subsequent in vivo corrosion in a rabbit model of 48 weeks.

**Fig. 1:**
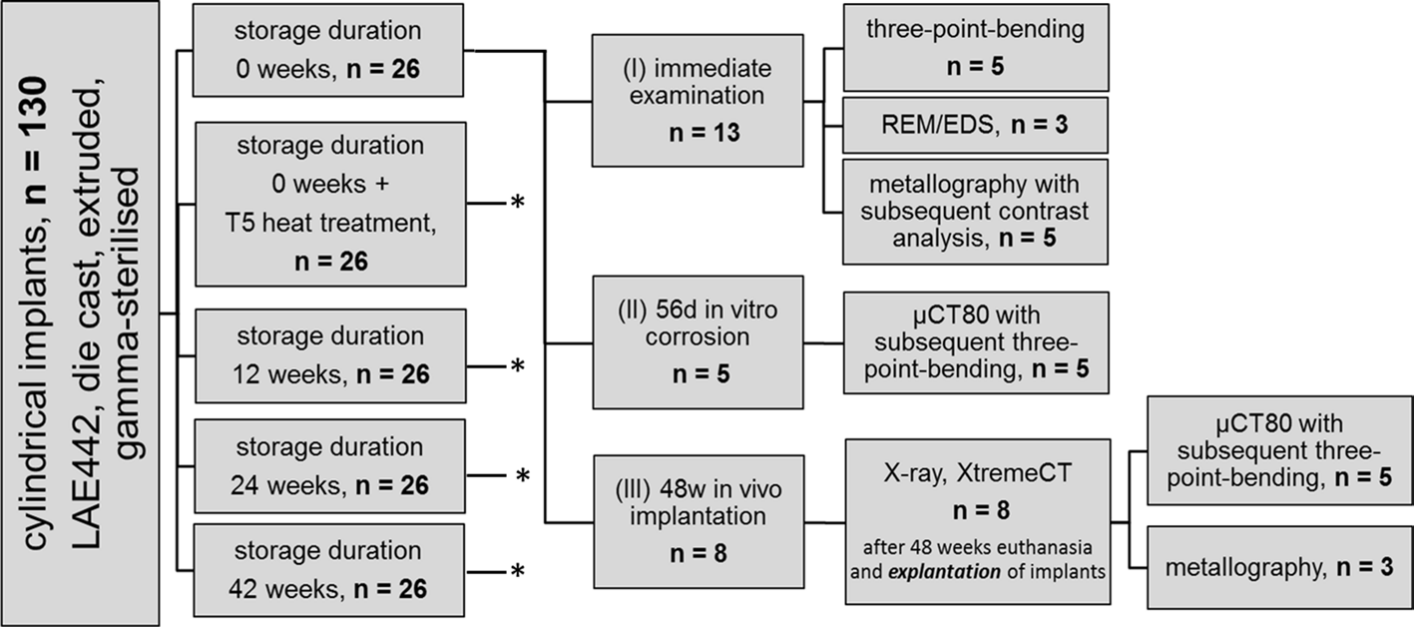
Flow chart of experimental set up. The complete experimental set up is shown for implants with no storage (storage duration 0 week). For all other groups the *asterisks* is representative for the exact execution of the three sub-studies (I, II, III) as in the group with 0 week storage duration

Figure 1 depicts the course of production and examinations.

### Experimental methods

#### Sub-study I: implant analysis after storage or heat treatment

Sub-study I dealt with the testing of the initial material directly after the respective storage period.

##### Three-point bending test

The mechanical properties of *five implants per group* were analyzed in a three-point bending test in accordance with DIN EN ISO 178 [15], as described by Krause et al. [16]. The bending punch moved downwards with a constant velocity of 1 mm/min. The abort criterion was a drop in force of 10 % or a bending punch displacement of 5 mm. The mean values of the maximum forces (F_max_ ([N])) of the different storage groups and the heat-treated group were recorded.

##### Scanning electron microscopy (SEM)

**Fig. 2:**
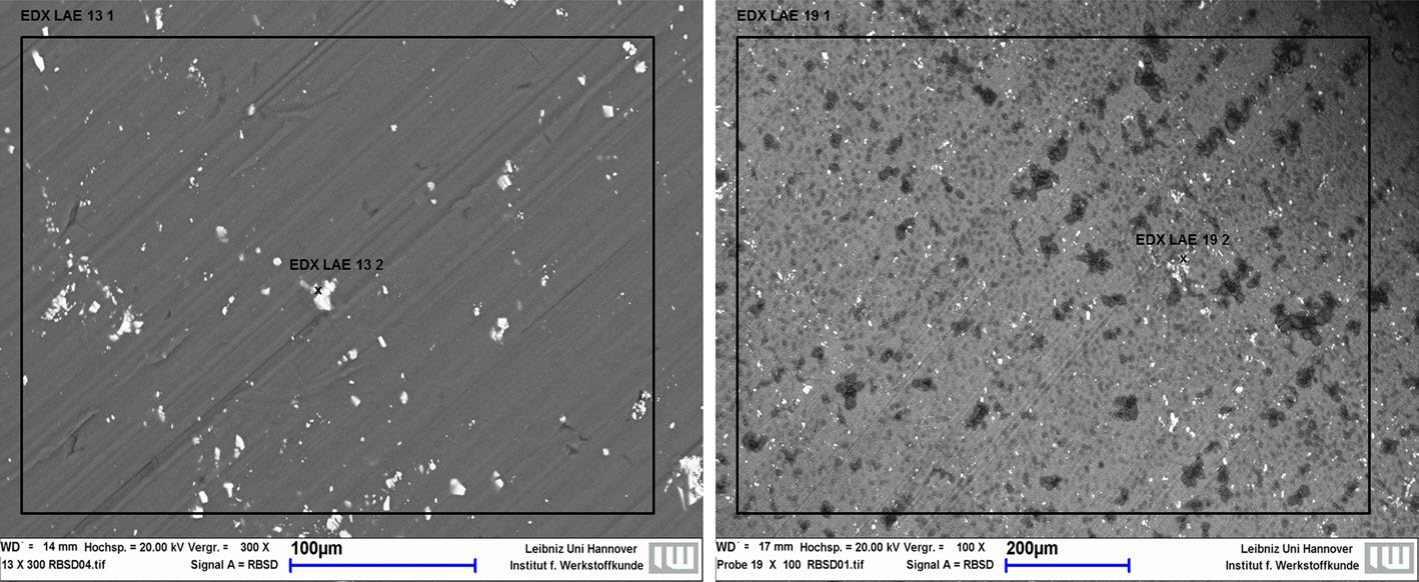
Exemplary SEM images of a defined area on the implant surface. The *black squares* curtailed the measuring area, the little cross marked a measuring point. Storage duration 0 week (*left*) and 48 weeks (*right*). The *black* points in the *right* image were oxygen enriched regions

A scanning electron microscope (SEM; LEO 1455VP, Zeiss, Oberkochen, Germany; resolution: 5 nm) with Rutherford Backscattering Spectroscopy (RBS) was used to characterize the surfaces of *three implants per group*. At selected areas of the implants' surface, an energy dispersive analysis (EDX; EDAX Genesis^®^, EDAX, Mahwah, USA) was performed to quantitatively determine the composition (Fig. 2). The results were computed with EDAX Genesis software and expressed as weight percent (wt%). Additionally, a descriptive assessment of the SEM images was performed.

##### Metallographical examination

In order to conduct metallographical analysis, *five implants per group* were embedded in a resin (Demotec 70; Demotec Metallografie, Nidderau, Germany) and subsequently treated with an etching solution (3 g picric acid, 20 ml acetic acid, 50 ml ethanol, 20 ml water). Lateral longitudinal, polished sections were prepared and examined to define the mean grain size in accordance with DIN EN ISO 643 [17]. It was calculated using the following equation [18]:

$${\text{K}}_{\text{mid}}\,=\,\sqrt {\frac{{{\text{A}}_{\text{Circle}} }}{{{\text{K}}_{\text{w}} { + }\frac{{{\text{K}}_{\text{c}} }}{ 2}}}}$$

whereas K_mid_ was defined as the average grain size, A_Circle_ as the area of the defined circle (5000 mm^2^) corresponding to a diameter of 79.8 mm, K_w_ as the number of whole grains in the circle, and K_c_ as the number of grains cut by the circle’s range. This calculation was repeated three times for each implant by three different individuals.

##### Contrast analysis

For contrast analysis, metallographic images showing precipitations were edited with Corel Draw^®^ (COREL™, Ottawa, Canada). Here the images were converted to black and white images using a constant threshold value. After conversion, black regions in the image correlated with the precipitations shown in the initial images. Subsequently the black/white ratio was determined using a script which was coded using MATLAB (The MathWorks©, Redmond, USA).

#### Sub-study II: in vitro corrosion after storage or heat treatment

To determine the effect of corrosion on the materials properties, *five implants per group* were stored in plastic tubes (101 × 16.5 mm) with simulated body fluid (SBF: 700 ml distilled water; 5.403 g NaCl; 0.504 g NaHCO_3_; 0.426 g Na_2_CO_3_; 0.426 g Na_2_CO_3_; 0.225 g KCl; 0.230 g K_2_HPO_4_ × 3H_2_O; 0.311 g MgCl_2_ × 6H_2_O; 100 ml 0.2 M—NaOH; 17.892 g HEPES; 0.293 g CaCl_2_; 0.072 g Na_2_SO_4_, pH 7.4, approx. 10 ml per tube) for 56 days at 37 °C. The temperature and pH were measured daily and SBF was changed when the pH exceeded a pH of 8.

##### µ-computed tomography (µCT80)

After 56 days of in vitro corrosion, the implants were scanned using a µ-computer tomograph (µCT80; ScancoMedical, Zurich, Swiss; slice thickness: 20 µm; voltage: 70 kV; amperage: 114 µA; integration time: 400 ms). 3D images were computed (threshold: 108) and an assessment of the volume, density and the “true-3D-thickness” of the implants according to Huehnerschulte et al. [19] was performed.

Subsequently, the samples underwent three-point-bending testing as described in “Three-point bending test”.

#### Sub-study III: in vivo degradation and biocompatibility after storage or heat treatment

Female, adult New Zealand White rabbits (n = 20, mean weight: 3.47 ± 0.45 kg; Charles River, Sulzfeld, Germany) were used for the animal experiments which were conducted according to the German federal welfare legislation (33.12.-42502-04-11/0640). The rabbits were housed separately in standard cages (Scanbur-BK, Karlslund, Denmark) as described previously [20].

##### Animal model

All animals were randomly divided into five groups each consisting of four rabbits. The LAE442 pins were implanted intramedullary in both tibiae. The anaesthesia method, surgery procedure, and medication have been described previously [21]. The follow up period covered 48 weeks.

##### In vivo analyses

Rabbits were examined clinically each day over the whole investigation period. The basic parameters of assessment were swelling, redness, wound dehiscence, appearance of pus, formation of emphysema, and accumulation of surrounding tissue hardness.

**Fig. 3:**
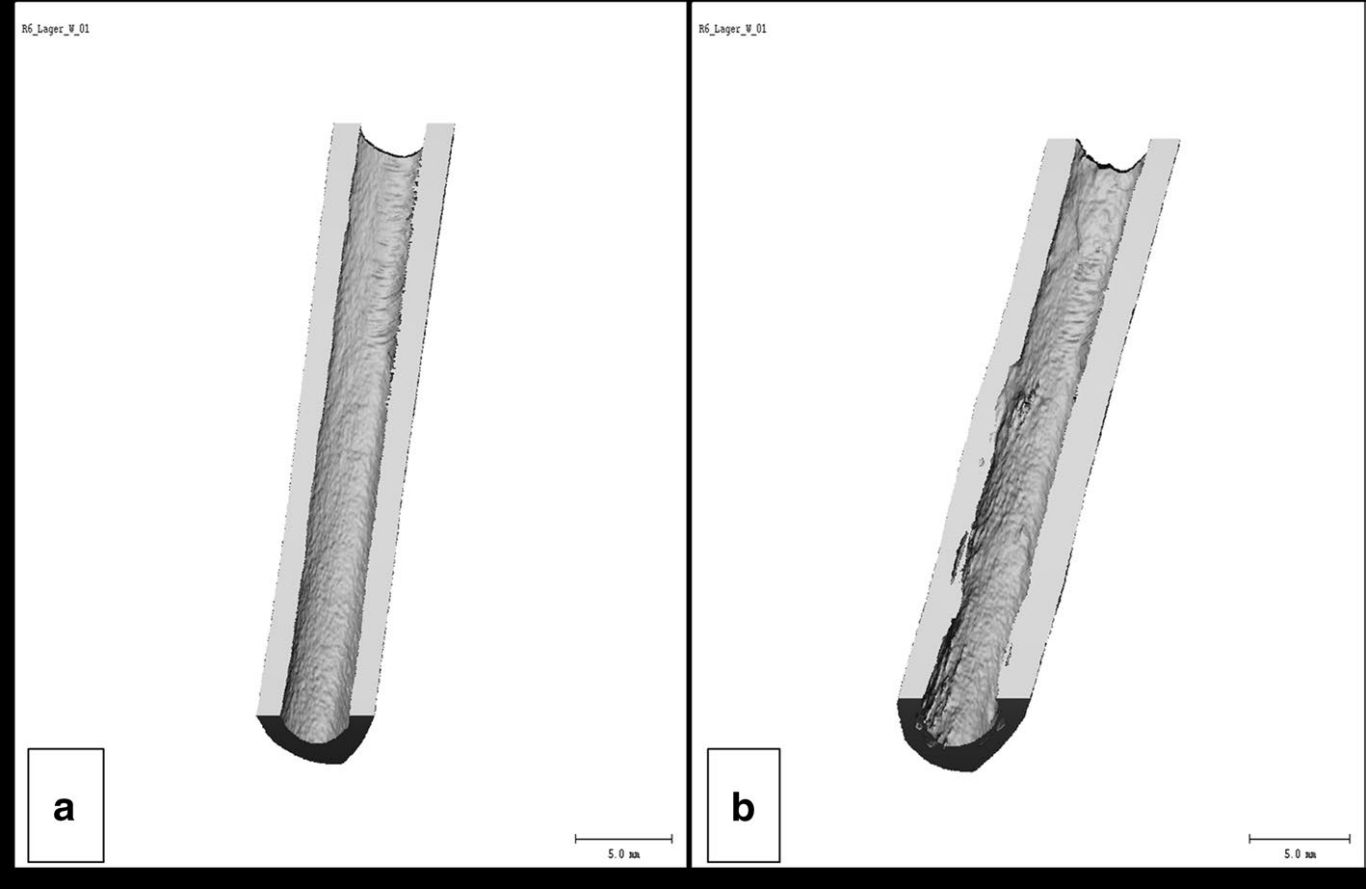
Exemplary 3D-images of the longitudinal cut bone of a rabbit. Storage duration of implant: 0 week, heat treated. The computed section of the tibia was defined by the implant location. **a** Scan immediately after implantation; **b** scan 48 weeks after implantation. In **b** the bony host response could be detected in form of irregular surface structures

Every 12th week, a µ-computed tomography (XtremeCT: ScancoMedical, Zurich, Swiss; slice thickness: 41 µm; voltage: 60 kV; amperage: 900 µA; integration time: 100 ms) was performed under general anaesthesia. After the computation and remodeling of each scan, the bone density, bone volume, and bone porosity were calculated, as well as implant density, volume, “true-3D-thickness” and a variance (Evaluation Program V 6.0: ScancoMedial, Zurich, Swiss, threshold bone: 160; threshold pin: 138) according to Huehnerschulte et al. [19] (Fig. 3).

**Fig. 4:**
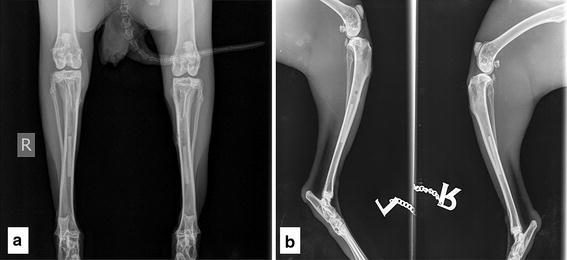
Exemplary X-ray images. Hind legs of a rabbit 4 weeks after surgery; implant storage duration 48 weeks: The pins were located in the middle third of the tibia; **a** anterior-posterior view; **b** mediolateral view. In both images the integrity of the implants is preserved, the cortices have an even structure and no gas formation could be observed

On each scanning day, medio-lateral and an anterior-posterior X-rays (film-focus-distance: 110 cm; 48 kV; 6.3 mAs) of the hind legs were taken (Fig. 4). Evaluation of these images was conducted in accordance with the semiquantitative score system used in previous studies [19, 20]. Thus, the analyses focused on bony growths at the implant location and diaphysis, accumulation of gas, and changes in the medullary cavity and corticalis using a four-point scale for the assessment (0, not pronounced to 3, strongly pronounced) [20]. The minimum and maximum values and the median were calculated.

##### Ex vivo analyses

Rabbits were euthanized at the end of the investigation period. Both tibiae were completely removed of the surrounding tissue. Furthermore the implants were removed by longitudinal cutting of the tibia (Dremel^®^ 300 Series, Dremel Europe, Leinfelden-Echterdingen, Germany). The *five of the explanted pins* were treated with chromic acid (200 g CrO_3_; 10 g AgNO_3_; 20 g Ba(NO_3_), 1000 ml distilled water) and scanned using the µCT80 (same as described in “μ-computed tomography (μCT80)”). Subsequently a three-point bending test was performed (see “Three-point bending test”). The remaining *three explanted pins* were prepared for metallographical examinations and the mean grain size was evaluated (see “Metallographical examination”).

#### Statistical analysis

The results of this study were analyzed using Microsoft^®^ Office Excel 2010 software (Microsoft Office XP, Microsoft Corporation, Redmond, USA) and SPSS^®^ version 21.0 (IBM Company, Chicago, USA). All groups were tested for normal distribution. In addition, a *t* test and an ANOVA with post hoc tests (Tukey or Games Howell) for the comparison of different groups were used. Non-normally distributed data were analyzed by Mann–Whitney U test or Wilcoxon W test. Results with p ≤ 0.05 and 0.01 were considered as significant and highly significant, respectively.

## Results

### Biomechanical properties

**Fig. 5:**
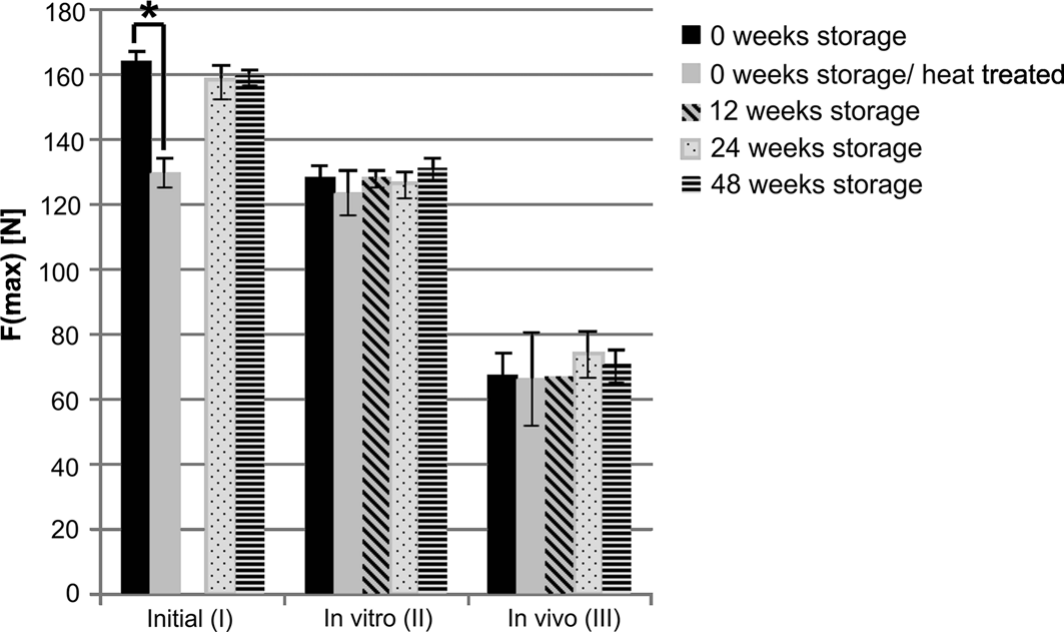
Results of the three-point-bending test. No significant differences between the different storage groups were found. Heat-treated pins showed a clearly lower initial stability (*statistically significant differences) but mechanical stability had adjusted to that of the untreated material after 56 days in vitro in simulated body fluid as well as after 48 weeks in vivo corrosion

There were no significant differences between the different storage groups in all three sub-studies. The mean maximum force of the implants decreased by 17.13 % after the in vitro immersion in SBF and by 55.25 % after implantation in vivo compared to the untreated samples. A clear difference (p ≤ 0.01) was observed between the heat-treated and the non-heat-treated implants after 0 week of storage tested immediately after the storage period (sub study I). No similar disparities occurred in the in vitro and in vivo part. All results of the three-point-bending tests are shown in Fig. 5.

### Structural properties

Evaluation immediately after the respective storing duration (sub-study I) showed that increasing storage duration resulted in a significant reduction in magnesium content on the implant surface, combined with an increase in oxygen-rich areas; heat treatment intensified these effects. In the SEM/EDX analysis, the magnesium content of pins stored up to 24 and 48 weeks was 68.30 wt% ± 1.39 and 62.86 wt% ± 3.82, respectively, and that of heat-treated pins was 71.73 wt% ± 0.16. Compared to not stored/not heat treated material (2.40 wt% ± 0.28) oxygen-rich areas increased after 24 (15.83 wt% ± 1.83) and 48 (17.27 wt% ± 2.42) weeks of storage, and after heat treatment (22.13 wt% ± 1.08). The percentage of aluminum was lower after 24 weeks (1.21 wt% ± 0.04) compared to 0 week of storage (1.64 wt% ± 0.51). The heat-treated group however showed the lowest amounts of aluminum (0.85 wt% ± 0.51). An increase of carbon with increasing storage duration and heat treatment (0 week: 3.44 wt% ± 0.29; 0 week/heat treated: 5.29 wt% ± 0.73; 24 weeks: 14.67 wt% ± 0.83; 48 weeks: 18.62 wt% ± 1.59 was determined.

**Fig. 6:**
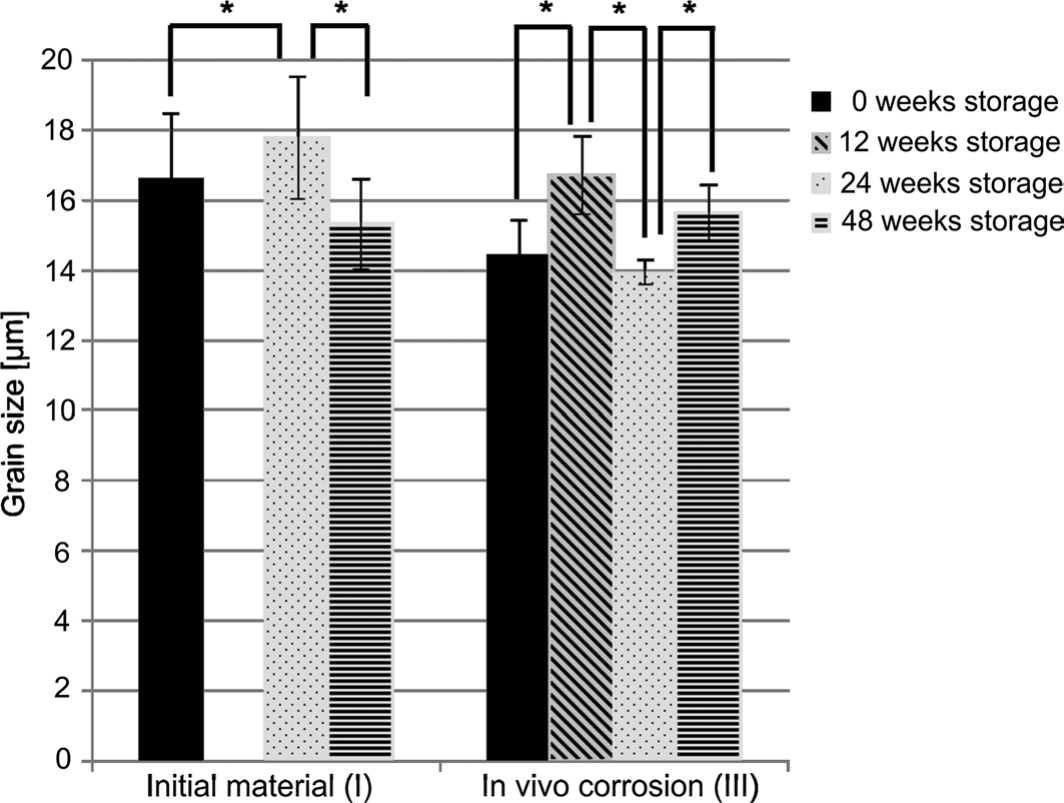
Results of the grain size analysis. The largest mean grain size in sub-study I was found for implants after 24 weeks storage duration (immediately after that period, sub-study I) and for implants after 12 weeks storage duration (subsequently to 48 weeks in vivo implantation, *statistically significant differences)

The mean grain size was evaluated immediately after storage (sub-study I) and after 48 weeks in vivo implantation (sub-study III) (Fig. 6). In sub-study I implants stored for 24 weeks had larger grains (17.79 µm ± 1.88) than all other groups with significant differences to the group stored for 0 week (16.59 µm ± 1.87) and 48 weeks (15.33 µm ± 1.47). In sub-study III implants stored for 24 weeks had the smallest mean grain size (13.95 µm ± 0.33) and those stored for 12 weeks had the largest mean grain size (16.72 µm ± 1.10) (Fig. 6).

**Fig. 7:**
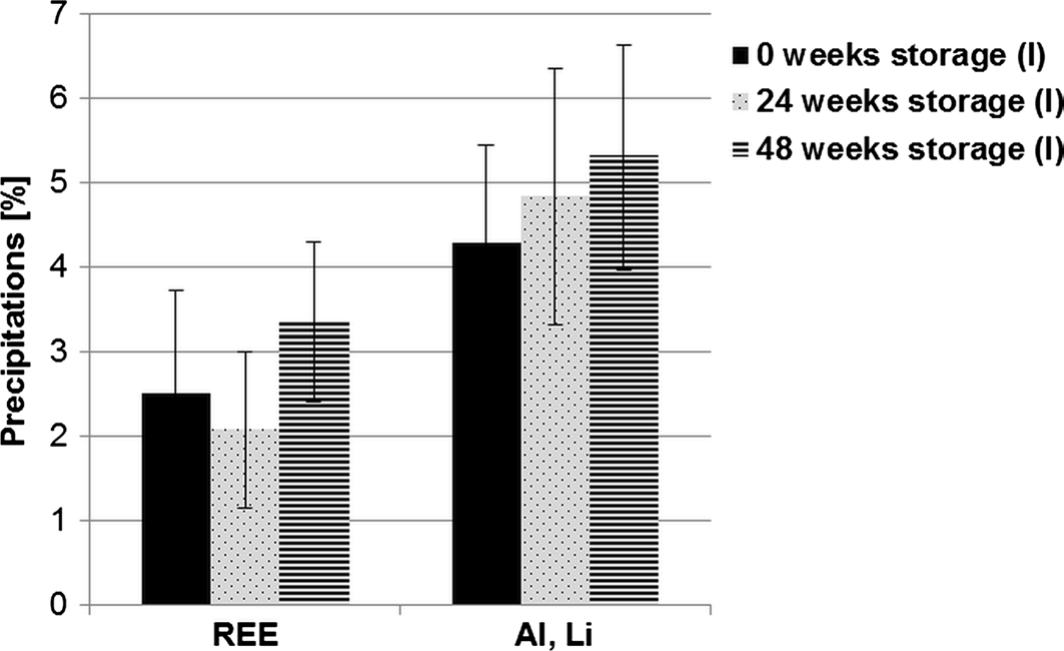
Results of the precipitation analysis immediately after storing (sub-study I). The highest amount of REE and Al, Li precipitations was observed after a storage duration of 48 weeks, but the differences were not significant related to the other storage groups

**Fig. 8:**
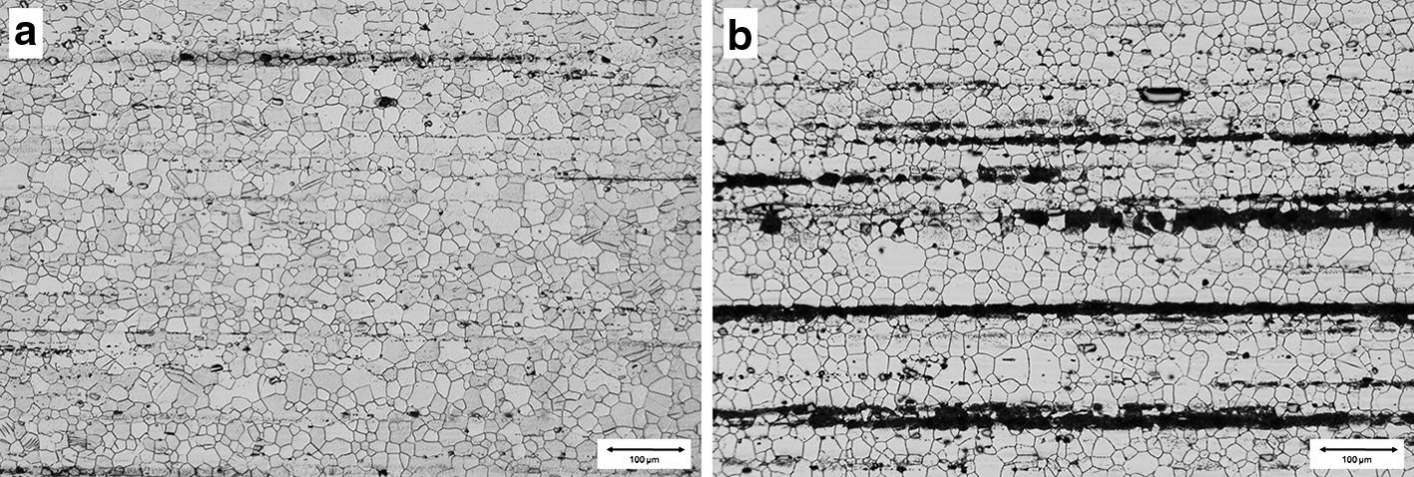
Metallographic images. Exemplary pictures of the microstructure of LAE 442 samples: **a** immediately after hot extrusion; and **b** immediately after hot extrusion and T5 heat treatment; polished and etched (picric and acetic acid, 20 %) sections longitudinal to the extrusion direction

In addition, contrast analysis evaluated the quantitative development of rare earth element precipitations and other precipitations such as aluminum and lithium. Here, no significant differences were observed between the different storage groups (Fig. 7). However, heat-treated implants showed high amounts of precipitations. Contrast analysis therefore was not considered as a reliable analysis method. Figure 8 shows the microstructure of as extruded (a) and as extruded and T5 treated (b) specimens directly after processing.

### µ-computed tomographical analysis of in vivo implant degradation

**Fig. 9:**
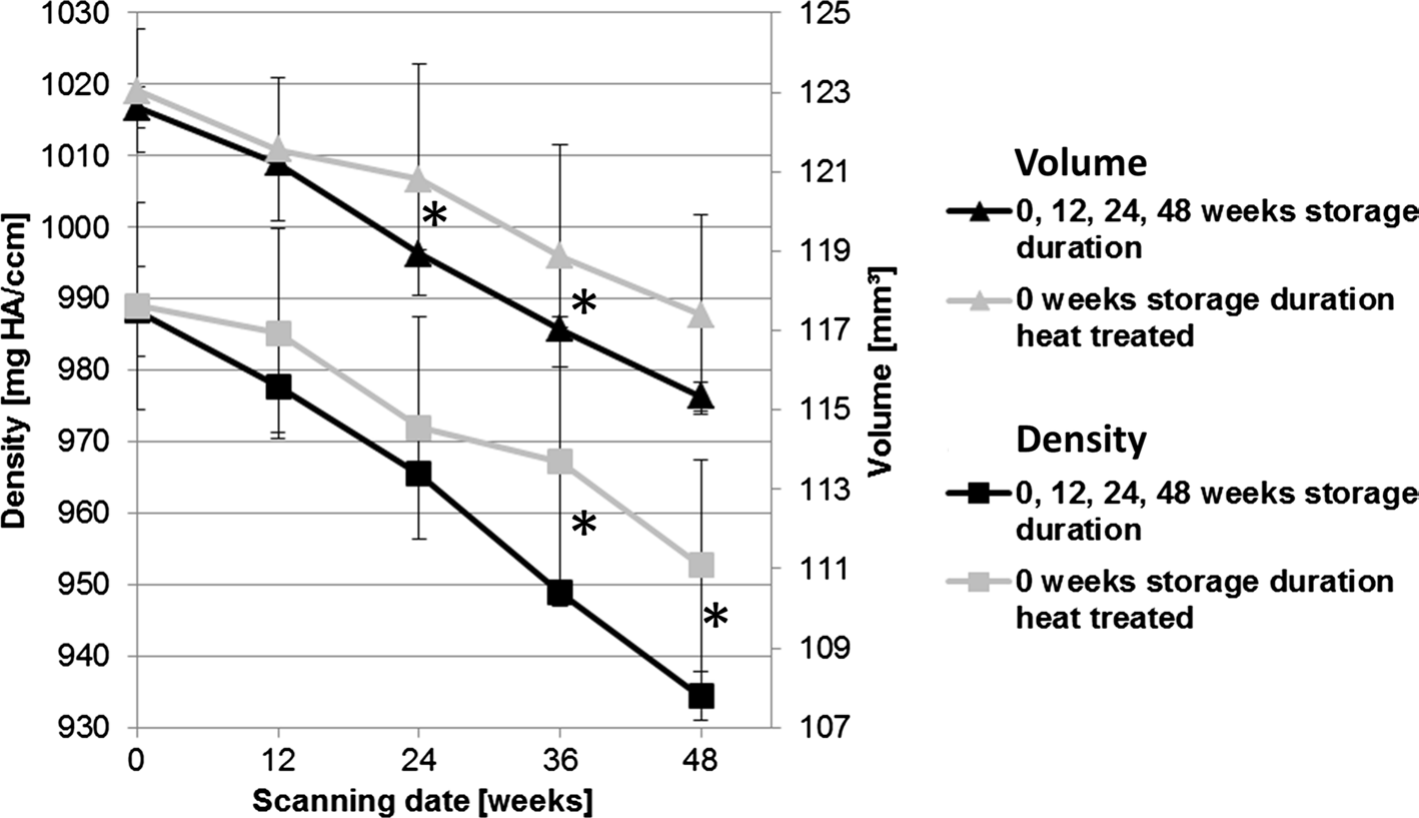
Comparison of implant volume and density over 48 weeks in vivo implantation. Storage groups without heat treatment were pooled and compared to heat treated implants with no further storage duration. All groups showed similar values at the beginning; however, in the final scan the group with the heat-treated implant showed higher values for both parameters (*statistically significant differences)

In the µ-computed tomography examination, data for pin density and volume between the different storage groups were normally distributed with low standard deviation. Since the results did not differ significantly after the different storage durations without further heat treatment, these results were pooled for a clearer presentation The pooled values were compared with those for heat-treated implants. In general, the heat-treated material showed a slower decrease in implant volume and density compared to the untreated groups. The results are represented in Fig. 9.

The “true-3D-thickness” decreased with increasing storage time in each group. The identified differences between the storage groups were detected by scanning at week 12 post-op [0 week storage (2.29 mm ± 0.24)–24 weeks storage (2.31 mm ± 0.19) (p = 0.036)], at week 36 post-op [0 week storage (2.16 mm ± 0.33)–0 week storage/heat treated (2.21 mm ± 0.36) (p = 0.037); 0 week storage/heat treated (2.21 mm ± 0.36)–48 weeks storage (2.16 mm ± 0.33) (p = 0.018)] and at week 48 post-op [0 week storage/heat treated (2.16 mm ± 0.34)–12 weeks storage(2.11 mm ± 0.35) (p = 0.047); 0 week storage/heat treated (2.16 mm ± 0.34)–48 weeks storage (2.10 mm ± 0.37) (p = 0.011)].

**Fig. 10:**
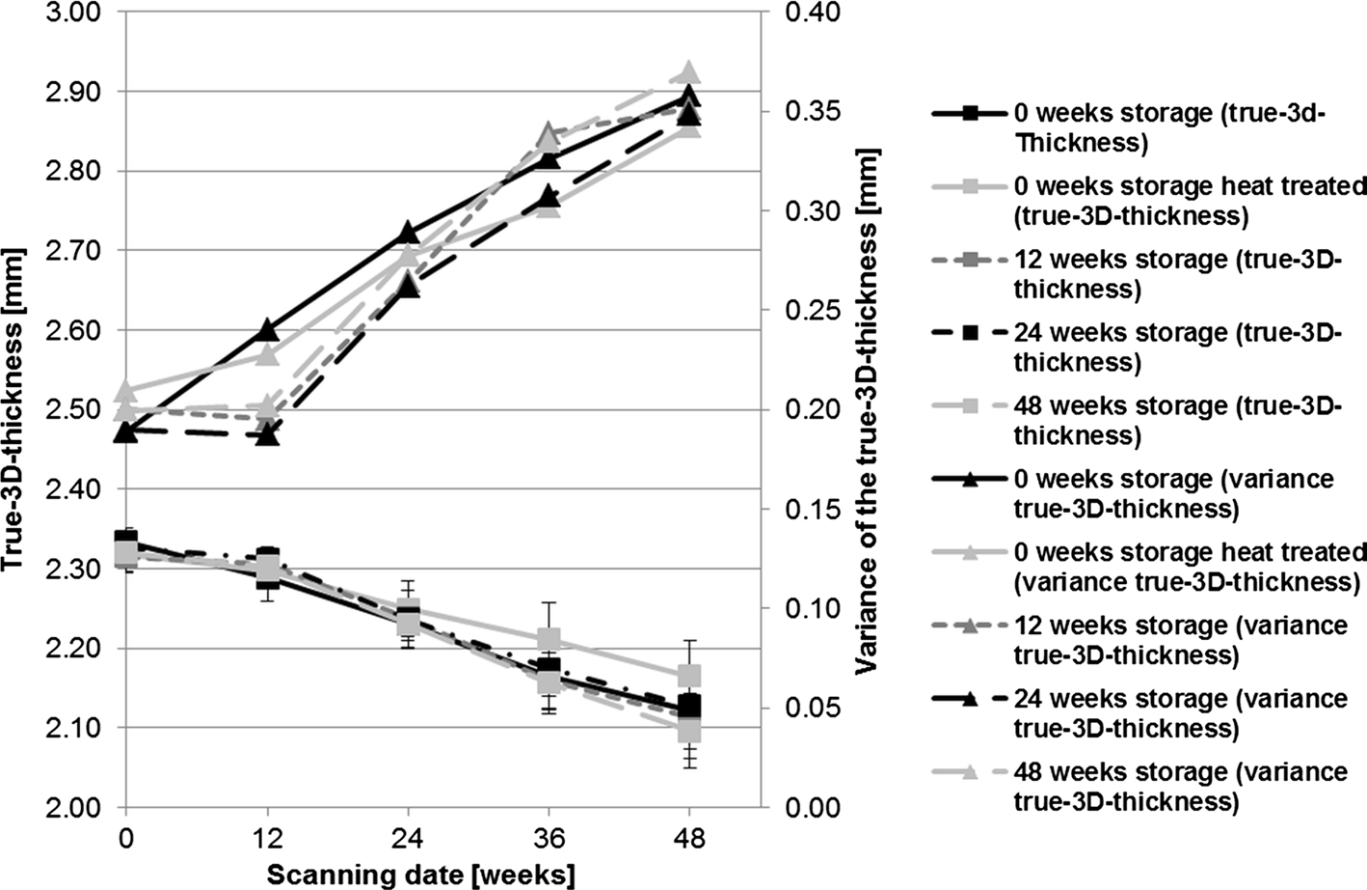
Results of the evaluation of the “true-3D-thickness” and its variance over the investigation period of 48 weeks. The “true-3D-thickness” decreased obviously 12 weeks after surgery in every storage group accompanied by an increase in variance at the same time point. Exception is the group after 0 week storage, which showed a rise of this parameter from scanning date 0

The variance of the “true-3D-thickness” increased obviously in all storage groups 12 weeks after surgery. The group with the directly implanted material (no prior storage) showed this increase in variance just from the beginning (Fig. 10).

#### Ex vivo µ-computed tomography of stored and heat-treated pins, and residual pins after in vivo implantation

**Fig. 11:**
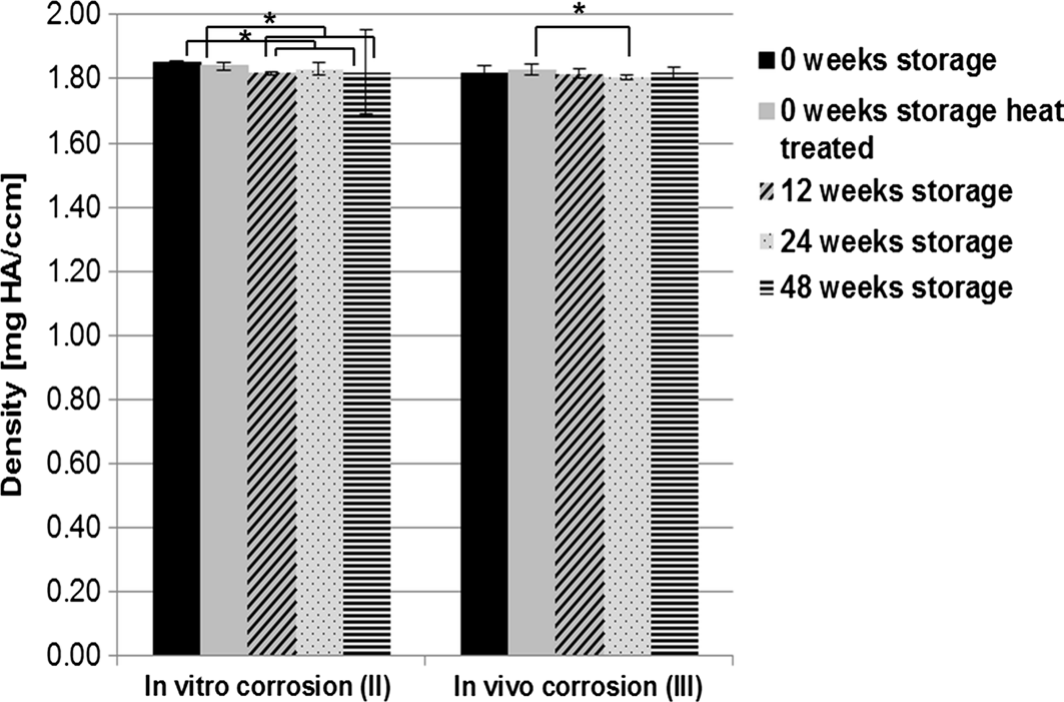
Results of µCT80 scans for the mean density (mg HA/ccm). All storage durations were evaluated after additional corrosion in SBF [“in vitro corrosion (II)”] and additional implantation in rabbit tibiae [“in vivo corrosion (III)”, *statistically significant differences]

**Fig. 12:**
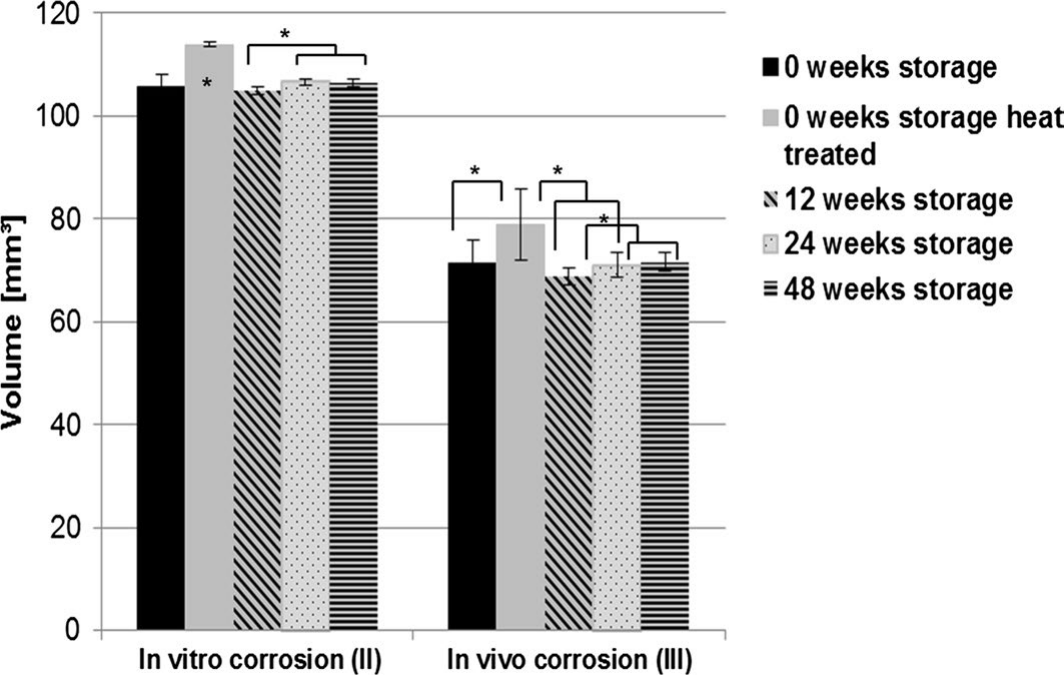
Results of µCT80 scans for the mean volume (mm^3^). All storage durations were evaluated after additional corrosion in SBF [“in vitro corrosion (II)”] and additional implantation in rabbit tibiae [“in vivo corrosion (III)”, *statistically significant differences]. Heat treated and not stored implants showed significant differences in volume after additional corrosion in SBF compared to every other storage group [“in vitro corrosion (II)”]

The additional scanning procedure of the explanted pins, using the higher resolution µ-computer tomograph (µCT80), showed higher values for density and volume of the heat-treated pins compared to the untreated pins (Figs. 11, 12). The µCT80 results of the implants after in vitro immersion showed that heat treated pins exhibit a significantly higher volume compared to every storage group. The density of these implants was also high, but the untreated pins without any storage showed the highest density results. Some clear differences between the different storage groups were detected for implant density and volume in both the in vitro and in vivo sub-study, but no trend could be observed.

### Biocompatibility

Slight inflammatory reactions such as swelling and redness in the region of the wound appeared in all groups in the first 3 weeks. None of the rabbits in any of the cohorts had any other clinical signs, with the exception of one animal (0 week of storage), which showed a light lameness in its left hind leg for 4 days 1 month after surgery. However, no clear abnormalities could be observed in an additional radiographic examination.

**Fig. 13:**
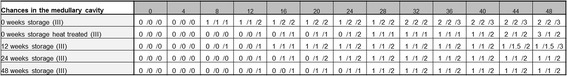
Evaluation of the X-ray images for changes in the medullary cavity. Development of the investigated parameter in all groups. Presented are the minimum/median/maximum scores at given time points

**Fig. 14:**
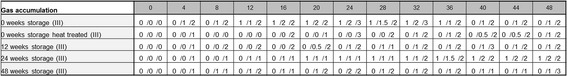
Evaluation of the X-ray images for gas accumulation. Development of the investigated parameter in all groups. Presented are the minimum/median/maximum scores at given time points

**Fig. 15:**
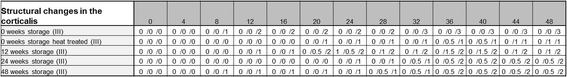
Evaluation of the X-ray images for structural changes in the corticalis. Development of the investigated parameter in all groups. Presented are the minimum/median/maximum scores at given time points

No formation of new bone on the diaphyses or the implantation side could be detected by X-ray. In every group, the cortical bone structure showed only small irregularities and the group with not stored implants did not show any conspicuity (Fig. 13). Accumulation of gas in the medullary cavity was also assessed. The animals with heat-treated implants exhibited the lowest formation of gas (Fig. 14). There were only small amounts of gas detected between week 32 and 48 of the investigation period. The groups with implants stored for 0 and 48 weeks showed a development of gas in weeks four and eight after surgery, with a peak (score 2) between weeks 16 and 32, and a score value of 1 for the remaining investigation time. The groups with the implants stored for 12 and 24 weeks showed small amounts of gas from week 20 until the day of euthanasia. The best results for hyperradiogenity of the medullary cavity adjacent to the implants were found for heat-treated implants (Fig. 15).

#### Postoperative in vivo µ-computed tomography

**Fig. 16:**
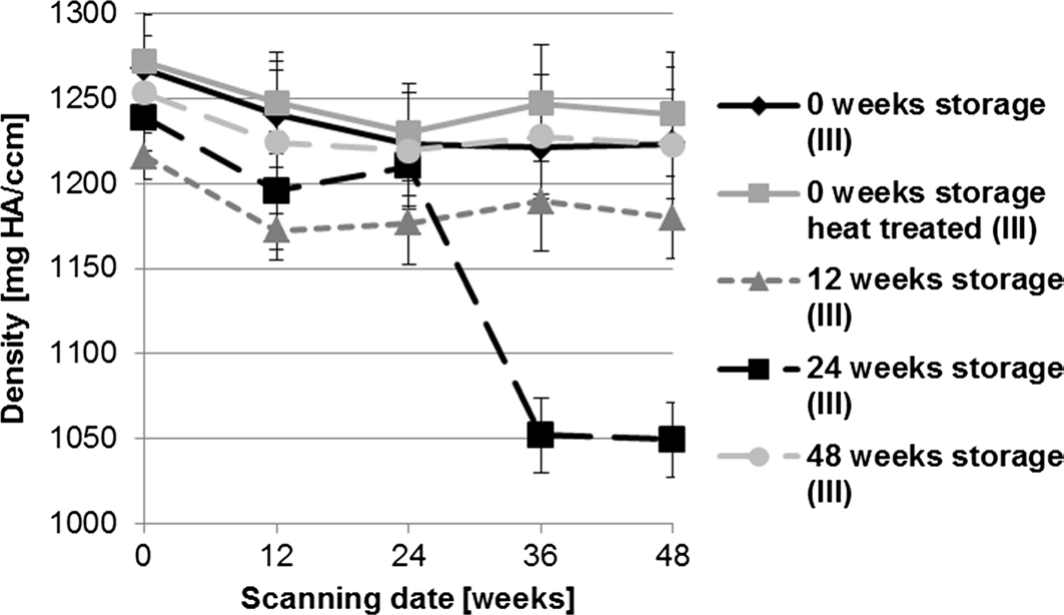
Development of the mean bone density evaluated by in vivo µCT (XtremeCT). Over the 48 weeks implantation period, density decreased in general in the first 24 weeks in every storage group in comparison to initial values. In the second half of the investigation period the mean density increased individually in the different groups

**Fig. 17:**
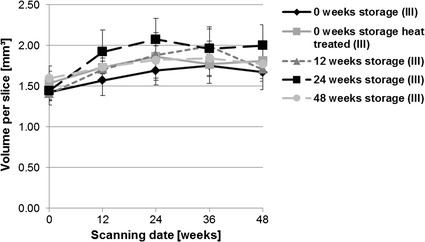
Development of the mean bone volume per slice evaluated by in vivo µCT (XtremeCT). Over the investigation period of 48 weeks the bone volume per slice increased obviously in the first 24 weeks in every storage group

**Fig. 18:**
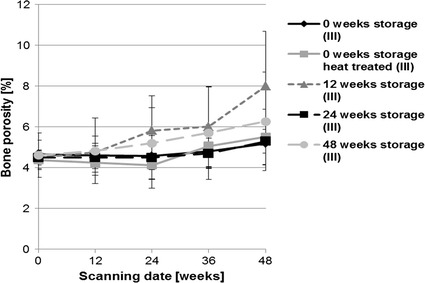
Development of the mean bone porosity evaluated by in vivo µCT (XtremeCT). Over the investigation period of 48 weeks bone porosity increased steadily from the beginning to the end of the implantation duration in every storage group

The host response in bone was evaluated by in vivo µ-computed tomography. Bone density significantly decreased during the first 24 weeks of postoperative follow-up (Fig. 16). However, no significant differences appeared between the storage and heat-treated groups. Bone volume between the different storage groups showed homogeneous increases (Fig. 17). At the beginning (scanning day 0) there were individual differences in volumes between storage conditions [0 vs 48 weeks storage (p = 0.025); 12 vs 48 weeks storage (p = 0.007)] but these reduced over the investigation period. An increase in bone volume was observed during the first 24 weeks in each group. The animals with implants stored for 24 weeks showed the highest bone volume over the whole period. Bone porosity significantly increased between weeks 36 and 48 in all groups (Fig. 18). The highest values for bone porosity were observed for 12-weeks stored implants after 48 weeks of implantation duration.

## Discussion

The purpose of this study was to investigate the influence of different storage durations and artificial aging caused by heat treatment on the degradable magnesium-based implant material LAE442.

In general, no significant influences of different storage durations of up to 48 weeks could be detected. This opposes the effect of artificial aging induced by heat treatment, which produced changes in both biomechanical and corrosive properties. Initial stability was significantly reduced after heat-treatment compared to the untreated material. However, this effect disappeared after the additional in vitro and in vivo corrosion where no significant differences between heat treated and non-heat treated samples could not be detected. A possible explanation is that a temperature around 200 °C might have impact on the structure of magnesium alloys which could be related to the initiation of recrystallization processes [22]. Similar findings have been described by Seitz [23] who stated that T5 heat treatments caused negative effects on the biomechanical properties of the alloy LAE442. Seitz [23] also observed a reduction in the maximum load until failure similar to the findings in the present study. A possible explanation might be that heat treatment promotes the precipitation of β-phases (Mg_17_Al_12_) or other brittle phases which influences mechanical [23–25] as well as corrosion properties [12]. Grain size is an important structural element in aging and was therefore analyzed in the present study. The size and arrangement of the grains shape the microstructure of an alloy and influence the characteristics of the material [26–30]. A uniform and small grain size is favorable, due to its positive impact on corrosion and mechanical behavior [13, 31]. Ullmann et al. [18] tested different material properties of the alloy LANd442, an alloy whose composition is similar to LAE442, after storage durations of one, three and 6 months. They reported that the grain size significantly decreased with increasing storage duration [18] although they could not provide an explanation for these findings. In the present study, a storage dependent clear change in grain size could not be observed when in vitro and in vivo results were considered. Whereas the smallest medium grain size of origin material was found after a storage duration of 48 weeks (15.33 µm ± 1.30; sub study I), implants stored for 24 weeks showed even smaller grain sizes when explanted after an in vivo period (13.95 µm ± 0.33; sub study III). However, the mean grain size in general was found to vary in a small range between 13.95 and 16.72 µm. These results indicate that the storage duration did not influence the grain microstructure to a relevant extent.

Confirming that no relevant changes of implant material occurred during storage, contrast analysis showed no effects on the development of precipitations (aluminum/lithium; rare earth elements).

However, storage did have an influence on the implant surface. During ongoing storage, a significant reduction in magnesium could be detected by SEM/EDX analysis, combined with an increase in oxygen-rich regions (24 weeks of storage increased this by a factor of 6.6 and 48 weeks of storage increased it by a factor of 1.1). Similar results were described by Ullmann et al. [7]. Most likely magnesium oxides or magnesium hydroxide layers formed on the samples surfaces during storage in this study, as it is known to occur on Mg surfaces under atmospheric conditions [32]. Longer storage duration can enhance the development of a protective corrosion layer.

The content of carbon on the implant surface also increased noticeably with increasing storage duration. The reason for this might be that hydrocarbon is transferred from the surrounding air into the surface layer of the implants. Another possible source of carbon could be the sterile polymer wrapping in which the samples were kept. The direct contact of the Polymer and the Mg surface could have caused a carbon transfer into the forming layers.

Although the oxide content increased indicating the formation of a protective corrosion layer, a significant influence on the corrosion behavior of the implants as analyzed by µ-CT evaluations could be assumed in vitro but was not observed in vivo. A significantly lower implant volume after in vitro corrosion was observed in the pins which were stored for 12 weeks compared with those stored for 24 and 48 weeks. The heat-treated pins showed the lowest volume reduction during in vitro corrosion and one of the highest values for density, which could be attributed to the formation of a more protective magnesium-oxide/hydroxide layer during the heat-treatment procedure, which furthermore correlates with the higher amounts of oxygen rich regions found by SEM/EDX analysis. Literature has shown that “grown” MgO films on the surface of magnesium specimens have a positive impact on the Mg’s corrosion resistance [33, 34]. It is expected that a T5-based heat treatment will have an impact on the alloys microstructure and the resulting phases which furthermore have an impact on the corrosion mechanisms the alloy will be impacted with. However, initially the samples will be completely covered by an oxide film that has a positive impact on the samples corrosion. After the oxide’s breakdown, precipitates will be exposed to the corrosive environment and possibly result in an altered corrosion behavior as in case of non heat treated LAE442. Therefore, while a T5 heat treatment procedure positively impacts the strength properties of an Mg alloy, it can, at the same time, negatively impact its corrosion properties. However, Song et al. prove that dense phase networks might result in positive impacts on a Mg alloy’s corrosion properties especially as nobler phase networks effectively shield the ignoble Mg matrix from the corrosive environment [32]. Within this study, however, it remains ambiguous if the T5 heat treatment has a significant impact on the corrosion properties of LAE442.

In the in vivo investigations, similar findings were observed for heat-treated pins, but no storage-related impacts could be observed. An advancing corrosion process demonstrated through the decreasing “3D-thickness” and an increasing standard deviation of the implant [19] was found in the 0-week storage group from the beginning of the study, and in all other groups from postoperative week 12. A slower corrosion process in heat-treated implants was confirmed by lower decreases in volume and density compared to the storage groups without heat treatment. A dense magnesium oxide layer resulting from the heat treatment might have enhanced the corrosion resistance [35].

Considering the influences of a heat treatment and storage on the biocompatibility of LAE442 implants, no clear differences could be observed in clinical investigations. The formation of gas is known to be a physiological process induced by the degradation of magnesium [36] and the excess of the physiological resorption capacity for this gas [37]. As no clinical signs of gas formation were observed in this study, the corrosion rate can be evaluated as acceptable. The unphysiological reaction of bone as another parameter for biocompatibility [38] was evaluated by µ-computed tomography. The determined increase in bone volume and decrease in bone density can be explained by remodeling processes. Until postoperative week 24 the bone volume increased due to new bone formation. Subsequently the bone volume decreased because the newly formed bony tissue had not mineralized completely. Here, Fuchs et al. [39] postulated that it takes 12 months to achieve a fully mineralized bone. The increase in bone porosity observed in the second half of the investigation period was the consequence of the appearance of cavities in the bone structure [20]. With respect to the different storage groups, there were individual differences at the beginning of the study in each of the three parameters (volume, density, and porosity), but this evened out over the 48 weeks of the study period, so that none of the storage groups showed a clear distinction compared to the other groups. To quantify the bone reactions histological analysis should be performed in another test series.

## Conclusion

The many evaluations carried out in the present in vitro and in vivo studies did not show a clear impact of standard storage procedures (dark, dry, room temperature) for up to 48 weeks on the biomechanical, structural, and corrosion properties of the degradable magnesium alloy LAE442. While storing the material, a natural ageing process could be observed as an increase in oxygen-enriched regions on the implant surface occurred. Nevertheless, the implants demonstrated an adequate degradation behavior and biocompatibility in vivo. By contrast, the T5 heat treatment produced a significant influence on the material characteristics by considerably reducing the initial stability while improving the corrosion resistance.

In summary, storage durations of up to 48 weeks are not critical for the degradable implant material LAE442 and heat treatment has to be used carefully depending on the favored application of the implant.

## Abbreviations

SBF: simulated body fluid
ICP-OES: inductively coupled plasma optical emission spectrometry
SEM: scanning electron microscopy
RBS: Rutherford Backscattering Spectroscopy
EDX: energy dispersive spectroscopy
µCT: µ-computed tomography
ANOVA: analysis of variance
LAE442: magnesium-based alloy with additionally up to 4 wt% lithium, up to 4 wt% aluminum and up to 2 wt% rare earth metals
LANd442: magnesium-based alloy with additionally up to 4 wt% lithium, up to 4 wt% aluminum and up to 2 wt% neodymium
AZ91D: magnesium-based alloy with additionally up to 9 wt% aluminum and up to 1 wt% zinc
AM60: magnesium-based alloy with additionally up to 6 wt% aluminum and below 1 wt% magnesium
